# Modeling congenital disease and inborn errors of development in *Drosophila melanogaster*

**DOI:** 10.1242/dmm.023564

**Published:** 2016-03-01

**Authors:** Matthew J. Moulton, Anthea Letsou

**Affiliations:** Department of Human Genetics, University of Utah, 15 North 2030 East, Room 5100, Salt Lake City, UT 84112-5330, USA

**Keywords:** *Drosophila*, Congenital disorders, Inborn errors of development, Fly models, Forward genetics, Reverse genetics

## Abstract

Fly models that faithfully recapitulate various aspects of human disease and human health-related biology are being used for research into disease diagnosis and prevention. Established and new genetic strategies in *Drosophila* have yielded numerous substantial successes in modeling congenital disorders or inborn errors of human development, as well as neurodegenerative disease and cancer. Moreover, although our ability to generate sequence datasets continues to outpace our ability to analyze these datasets, the development of high-throughput analysis platforms in *Drosophila* has provided access through the bottleneck in the identification of disease gene candidates. In this Review, we describe both the traditional and newer methods that are facilitating the incorporation of *Drosophila* into the human disease discovery process, with a focus on the models that have enhanced our understanding of human developmental disorders and congenital disease. Enviable features of the *Drosophila* experimental system, which make it particularly useful in facilitating the much anticipated move from genotype to phenotype (understanding and predicting phenotypes directly from the primary DNA sequence), include its genetic tractability, the low cost for high-throughput discovery, and a genome and underlying biology that are highly evolutionarily conserved. In embracing the fly in the human disease-gene discovery process, we can expect to speed up and reduce the cost of this process, allowing experimental scales that are not feasible and/or would be too costly in higher eukaryotes.

## Abstract (precis)

***Drosophila* Collection:** This Review highlights several *Drosophila* models of human inborn errors of development, with particular emphasis on the strategies geneticists have developed to break through the current genotype-to-phenotype bottleneck.

## Introduction

Congenital anomalies, or conditions that are manifest at or before birth, affect 3% of newborns in the USA (Kochanek et al., 2012; CDC, 2008) and 6% of newborns worldwide (Christianson et al., 2006). Many of these conditions are caused by heritable mutations, although environmental factors can also cause and/or contribute to the incidence and severity of congenital anomalies. In far too many cases, congenital disorders cannot be fully abrogated, accounting for 7% of all deaths among children under age 5 worldwide – more than the mortality due to HIV/AIDS and measles in this age group combined (Mathews et al., 2015). This percentage is much higher in the USA (20%) and in Europe (25%) (CDC, 2008; Kochanek et al., 2012; Liu et al., 2015). Syndromic congenital disorders, which manifest numerous simultaneous defects and account for about half of all cases of congenital anomaly at birth (Winter, 1996), are particularly difficult to manage clinically {e.g. CHARGE syndrome manifesting **coloboma** [emboldened words and phrases are defined in the glossary (see Box 1)], heart defects, **choanal atresia**, growth retardation, genitourinary malformation and ear abnormalities (Hsu et al., 2014), and velocardiofacial or Shprinstzens syndrome manifesting cardiac anomaly, **velopharyngeal insufficiency**, aberrant calcium metabolism and immune dysfunction (Chinnadurai and Goudy, 2012)}. Estimates suggest that the cause of at least 50% of congenital abnormalities remains unknown (Lobo and Zhaurova, 2008). It is vital that we understand the etiology of congenital anomalies because this knowledge provides a foundation for improved diagnostics as well as the design of preventatives and therapeutics that can effectively alleviate or abolish the effects of disease.

**Box 1. Glossary**

**Amorphic/hypomorphic allele:** an allele with complete (amorphic) or partial (hypomorphic) loss of function of a gene.

**Anophthalmia/microphthalmia:** a condition in which formation of the eye is completely (anophthalmia) or severely (microphthalmia) abrogated.

**Bicuspid aortic valve disease:** a congenital condition in which two of the leaflets of the aortic valve are fused, forming a bicuspid valve instead of a tricuspid valve.

**Brachydactyly:** a condition characterized by shortening of the digits.

**Cerebral autosomal-dominant arteriopathy with subcortical infarcts and leukoencephalopathy (CADASIL):** a hereditary disorder that affects blood flow in blood vessels (often in the brain), resulting in strokes, migraine, recurrent seizures and white-matter deterioration.

**Choanal atresia:** a congenital disorder in which the back of the nasal cavity (choana) is blocked by tissue remaining after incomplete recanalization of the nasal fossae.

**Coloboma:** a congenital defect resulting in a hole in an eye structure (especially the iris).

**Epifluorescence:** visualization of an object in an optical microscope by excitation of a fluorophore incorporated into the sample. Light radiation given off from the viewing side excites the fluor and reflected light is captured as the image.

**Epistasis:** genetic interaction of non-allelic mutations that mask the phenotype of other mutations.

**Gene regulatory network (GRN):** a set of interacting genes working in coordination to alter gene expression.

**Genetic redundancy:** genetically distinct but functionally similar gene duplicates usually arising from paralogous gene duplication. Loss of any gene might not result in an overt phenotype if similar genes with redundant function can function in place of the lost gene.

**Homeodomain transcription factor:** a protein containing a domain that physically interacts with a DNA molecule and activates transcription nearby.

**Imaginal disc:** any portion of the *Drosophila* larval epidermis that will give rise to a particular organ after metamorphosis. There are 15 imaginal discs in the fly, which give rise to the wing, eye, leg, etc.

**Infantile myofibromatosis-2 (IMF2):** a congenital disorder characterized by aberrant mesenchymal cell proliferation resulting in benign skin, muscle, bone and visceral tumors.

**Lateral meningocele syndrome (LMNS):** a congenital disorder manifest as distinctive facial features, hypotonia, hyperextensibility, and neurological dysfunction due to protrusion of the meninges of the brain or spinal cord resulting from a defect in the cranium or spinal column.

**Leukodystrophy:** a disease characterized by degeneration of the white matter of the brain.

**Orphan human disease:** a disease that affects a relatively small population (generally <200,000 affected people in the USA), for which there is little or no therapeutic intervention available.

**RAS/MAPK pathway:** signaling pathway in which an extracellular signal peptide binds to a membrane-bound receptor and activates an intracellular signaling cascade involving RAS protein, which activates MAP kinases (MAPKs). The signaling cascade culminates in the activation of a transcription factor, which initiates transcription of a set of target genes.

**RASopathy:** family of diseases caused by mutations in RAS/MAPK signaling pathway components.

**Spondylocostal dysostosis:** a group of disorders of the axial skeleton characterized by a reduced rib number as well as defects in vertebra alignment and rib alignment.

**Velopharyngeal insufficiency (VPI):** a congenital disorder associated with an improper closing of the soft palate muscle (velopharyngeal sphincter) resulting in air escape through the nose instead of the mouth during speech.

One of the most fruitful ways to understand human congenital anomalies and to discover prophylactic treatments is to study them in animal models. The high degree of conservation of fundamental biological processes between humans and the fruit fly *Drosophila melanogaster*, coupled with the broad repertoire of genetic approaches to which *Drosophila* is amenable, make this organism a uniquely powerful model system for understanding the basic biological etiology of human disease and development (Bier, 2005; Pandey and Nichols, 2011; Ugur et al., 2016). Comparisons of the *Drosophila* and human genomes reveal a very high level of conservation (Adams et al., 2000; Lander et al., 2001; Venter et al., 2001). Overall, homologous fly and human proteins share about 40% sequence identity; this increases to 80-90% or higher in conserved functional domains (Rubin et al., 2000). Importantly, 75% of human disease-related genes are thought to have a functional homolog in *Drosophila* (Chien et al., 2002; Reiter et al., 2001).

Detailed analysis has revealed the *Drosophila* genome to be far less complex than the human genome (Hartl, 2000). Indeed, it is the simplicity of this genome that in large part accounts for the fly's genetic tractability. *Drosophila* has about 14,000 genes on four chromosomes; three of these chromosomes account for 96% of the animal's genome (Adams et al., 2000). In comparison to humans, the fly has about 1/20 as much DNA, 1/8 as many chromosomes and 1/2 as many genes (Lander et al., 2001; Venter et al., 2001). The fly also has fewer gene duplications, with those in the human genome resulting from large-scale DNA duplications in an early chordate ancestor 350- to 650-million years ago (Bell et al., 2009; McLysaght et al., 2002). These characteristics make the fly a highly genetically tractable organism. Additional features of the fly that make it an accessible model to work with include: its rapid generation time (8.5 days under ideal conditions at 25°C); large family size (a single mating fly pair produces hundreds of genetically identical progeny within days); and small size (hundreds of flies can be housed in a single 6 oz polyethylene bottle) (Ashburner et al., 2011; Ashburner and Thompson, 1978). Each of these features contributes to a substantially lower cost for fly husbandry in comparison to other animal models, permitting experimental scales not feasible in most other experimental models.

At the organismal level, the adult fly is complex and not unlike humans. The fly has structures equivalent to the human heart, lung, liver, kidney, gut, reproductive tract and brain (Behr, 2010; Jeibmann and Paulus, 2009; Lesch and Page, 2012; Roeder et al., 2012; Wolf and Rockman, 2008; Ugur et al., 2016). The fly brain consists of more than 100,000 neurons, which form elaborate circuits governing insect behavioral processes such as locomotion, circadian rhythms, mating, aggression and feeding (Simpson, 2009). The visual system of the adult provides an exceptionally rich experimental system, yielding key information about vision as well as development (Baker et al., 2014; Borst and Helmstaedter, 2015; Paulk et al., 2013; Wernet et al., 2014). A landmark study by the late Walter Gehring revealed the fly and human eyes to be homologous structures (Halder et al., 1995). Products of divergent (rather than, as long thought, convergent) evolution, both the fly and human eye are dependent upon *Pax6* for their development (Gehring and Ikeo, 1999), and the two share an evolutionary ancestor – a marine rag-worm, *Platynereis* (Arendt et al., 2004).

**Fig. 1.**
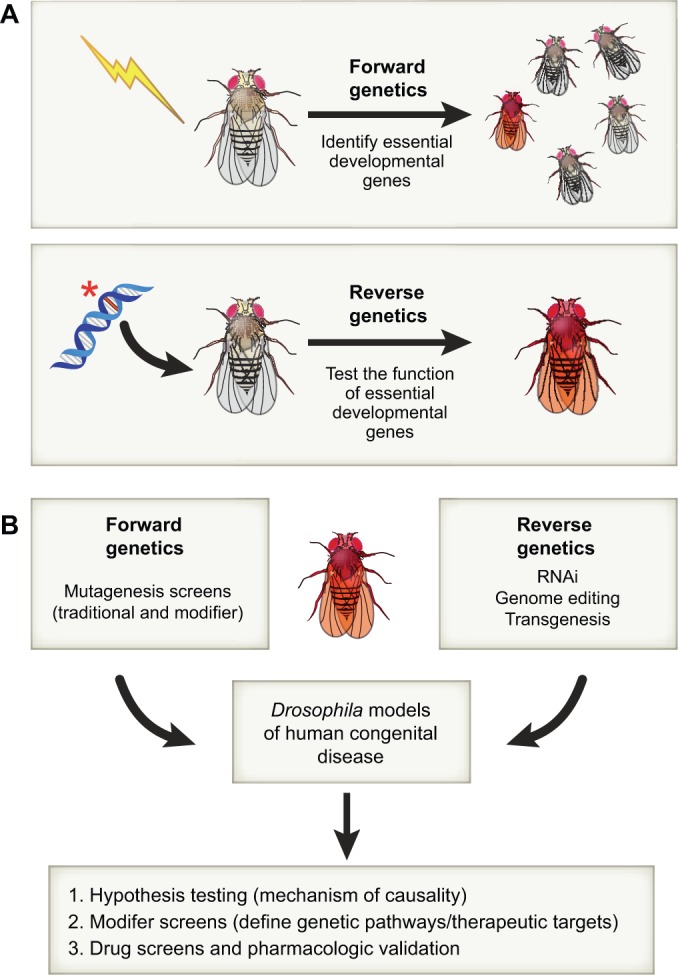
**Forward and reverse genetic approaches in *Drosophila*.** (A) Forward genetics uncovers the genetic basis of phenotype. Mutagenesis by any means (e.g. X-rays, chemicals or transposons; indicated by a lightning bolt) is used to generate mutant flies with aberrant phenotypes (indicated by the red fly), which are used as a starting point for gene discovery. Reverse genetics refers to the discovery of gene function through the targeted disruption of genes (here indicated by an asterisk showing a mutation in a gene sequence) and the analysis of the resulting phenotype(s). (B) Both forward and reverse genetic strategies are useful for the creation of animal models of disease that can be used as platforms to test hypotheses, perform modifier screens and identify new therapeutics. (A,B) In both panels, wild-type flies are shown in brown, mutant flies in red.

Here, we explore the methods that have proven successful in generating *Drosophila* models for human congenital disorders. We discuss both forward and reverse genetic approaches (Fig. 1, Box 2), noting that, when the first genetic screens were undertaken in experimental systems such as *Drosophila* and *C. elegans*, the depth of the genetic homology shared between these organisms and humans was not yet evident. We highlight how outcomes from these screens yielded mechanistic details of signal transduction and shed light on the etiology of human congenital disorders affected by these pathways. Later, with the emergence of universal rules for metazoan development, forward genetic methods were employed to enhance our understanding of developmental programs in tissues and organs dependent upon conserved core regulatory networks for their growth and elaboration. Now, with the advent of the post-genomic age, investigators have turned to reverse genetic methods to directly assess roles of human disease gene candidates via gene knockdown/knockout and transgenesis, as described in the final section. Throughout, we focus on examples of *Drosophila* models of human inborn errors of development that have led to insights into etiology and which have informed the design of preventative and therapeutic treatment strategies.

**Box 2. Genetic methodologies**

**ΦC31-mediated transgenesis:** method of inducing integration of an injected plasmid at a specific site in the genome. An integrase protein, ΦC31, induces recombination between the bacterial attachment site (*attB*) in the injected plasmid and the phage attachment site (*attP*) in the genome.

**CRISPR/Cas9:** method of inducing targeted double-stranded breaks in the genome. CAS9 binds to RNA (termed guide RNA) that pairs with genomic DNA and induces a double-stranded DNA break. Improper repair at these breakpoints in cells that give rise to the germline can lead to mutations that can be isolated in the next generation. Additionally, double-stranded DNA breaks can induce the incorporation of foreign DNA containing homology arms surrounding the break point. This system has been utilized to generate novel mutations in genes as well as facilitate targeted knock-in strategies.

**Forward genetic screen:** random, genome-wide mutagenesis to generate progeny with an aberrant phenotype(s). Identification of individual mutated genes leads to the discovery of genes involved in any given process. Identification of different genes with shared loss-of-function phenotypes leads to the discovery of genetic pathways. Traditional forward genetic screens in *Drosophila* using X-ray, chemical and transposon mutagenesis have uncovered numerous genetic pathways involved in development. These pathways and their function in development are often conserved in humans.

**GeneSwitch:** method to control induction of gene expression spatially and temporally. This method utilizes a GAL4–progesterone-receptor chimera protein that can be activated by the hormone progesterone.

**Modifier screen:** random mutagenesis performed in a mutant background (usually hypomorphic) to identify mutations that enhance or suppress a mutant phenotype. Modifier screens yield additional genes involved in a given process/pathway, including both integral and modulatory pathway components.

**Mosaic analysis with a repressible cell marker (MARCM):** system to generate labeled mutant mitotic clones within a field of wild-type cells. This system requires the use of: (1) the inducible gene expression system in which GAL4 protein activates transcription at upstream activation sites (UAS), (2) the repressor of GAL4 induction, GAL80, (3) spatiotemporally controlled expression of the DNA recombinase Flipase (FLP), and (4) a marker (usually fluorescent) downstream of the UAS. The mutant allele of interest and the *GAL80* transgene are recombined onto homologous chromosome arms containing FRT sites (the site at which FLP-mediated recombination will occur). FLP-induced mitotic recombination in cells heterozygous for the *GAL80* transgene and the mutant allele of interest yields recombinant daughter cells that inherit either two copies of the mutant allele or two copies of the *GAL80* transgene. Daughter cells lacking GAL80 and harboring the homozygous mutant allele will express the marker in a field of unmarked cells that did not undergo recombination or are homozygous for GAL80.

**Reverse genetic screen:** targeted mutagenesis of any given gene designed to understand the gene's biological function. Mutagenesis can be accomplished via numerous mechanisms, such as RNAi or CRISPR/Cas9.

**RNA interference (RNAi):** method of depleting a cell of a specific target mRNA. This is typically accomplished by expressing cytoplasmic double-stranded RNA that is subsequently processed by the cell into small single-stranded RNA molecules that are then used as templates to target and degrade complementary mRNA in the cell.

**Temporal and regional gene expression targeting** (**TARGET**): method to control induction of gene expression spatially and temporally. This method utilizes the UAS/GAL4 system in conjunction with a temperature-sensitive GAL80 to repress GAL4 activity at permissive temperatures.

## Models of human congenital disorders and inborn errors of development

*Drosophila* has a rich experimental history in genetics and development, beginning with the observation that genes are organized on chromosomes and leading to Thomas Hunt Morgan's 1933 Nobel Prize in Medicine (http://www.nobelprize.org/nobel_prizes/medicine/laureates/1933/). Later in the 20th century, burgeoning molecular genetic analyses thrust *Drosophila* into a new age of discovery by enabling systematic spatiotemporal control of transgenes (Rubin and Spradling, 1983), initially through the use of the UAS:GAL4 (Brand and Perrimon, 1993) and FLP:FRT (Golic, 1994) gene regulatory systems, and most recently through gene-knockout and gene-editing strategies (Beumer and Carroll, 2014; Boutros and Ahringer, 2008; Gong and Golic, 2003). Together, these methodological breakthroughs, along with their second-generation reinventions [e.g. MARCM (Wu and Luo, 2006), TARGET (McGuire et al., 2003), GeneSwitch (Nicholson et al., 2008; Osterwalder et al., 2001) and ΦC31-mediated transgenesis (Groth et al., 2004); Box 2], have yielded a richness of information that illuminates the principles and rules by which gene products and cells interact with one another to control development, with implications for understanding disease.

### Forward genetics – defining pathways and associated dysmorphologies

Forward genetic analysis (see Fig. 1) is an unbiased method for identifying gene function and is one of the most powerful approaches for understanding the genetic basis of human development and disease. Its impact on understanding the genetic basis of human development was first illustrated by the Nobel-Prize-winning screen pioneered by *Drosophila* geneticists Christiane Nusslein-Volhard and Eric Wieschaus (Roush, 1995). Their genome-wide screens for mutations that affect the pattern of the *Drosophila* cuticle led to the discovery of hundreds of loci that have essential and conserved roles in development (e.g. Nusslein-Volhard and Wieschaus, 1980). Complementing these elegant yet traditional screening endeavors were a subsequent generation of modifier screens (both enhancer and suppressor; e.g. Rogge et al., 1995; Box 2) that revealed not only genes encoding products that function as essential components of signaling pathways but also those that play modulatory roles. Most of the loci identified in these screens are conserved and encode comparable functions throughout metazoan lineages, including that of humans (Rubin et al., 2000). Indeed, the Heidelberg screens, which relied on female sterility and cuticle phenotypes for high-throughput screening, successfully yielded key components of several essential developmental signaling pathways, such as the Toll (Tl), Decapentaplegic (Dpp), Hedgehog (Hh), Notch (N), Fibroblast growth factor (FGF), Wingless (Wg), Engrailed (En) and Hippo (Hpo) pathways. The use of forward genetic screens in *Drosophila* has led to substantial insights into the cellular and molecular basis of processes that can go awry in development (Table 1), a few examples of which are highlighted below.

**Table 1.**
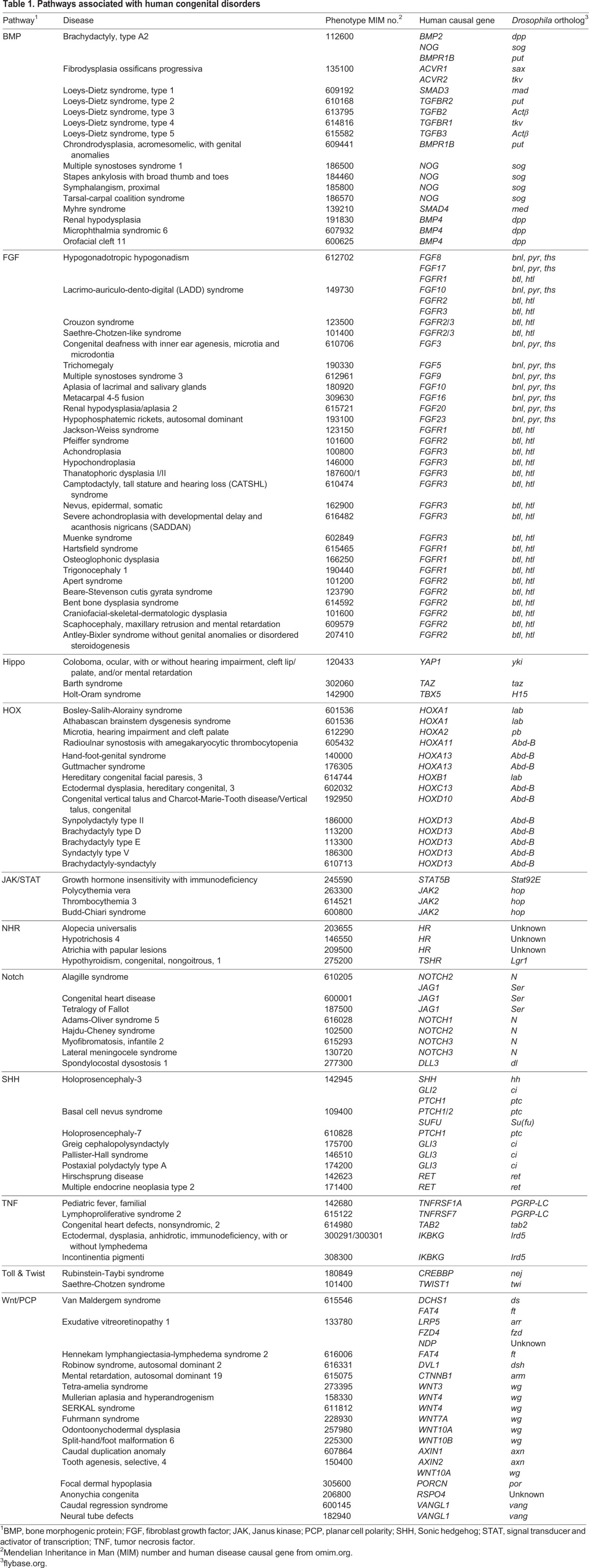
**Pathways associated with human congenital disorders**

#### The Toll pathway

Although perhaps best recognized for its conserved role in innate immunity, the Toll pathway, along with the CREB-binding protein (CBP) cofactor (called Nejire in *Drosophila*), modulates the activity of the Twist transcription factor via activation of NFκB [Nuclear factor κB; a transcription factor called Dorsal (Dl) in *Drosophila*] in early development in both flies and humans (Akimaru et al., 1997; Petrij et al., 2008; Wasserman, 2000). Reduced expression of Twist disrupts embryonic mesoderm differentiation in all metazoa (Castanon and Baylies, 2002). In humans, reduced expression of Twist (caused either by loss of a single copy of *CBP* or by hemizygosity for *Twist* itself) manifests as genetically related autosomal dominant developmental syndromes, either the rare syndrome Rubinstein-Taybi (1:100,000-1:125,000 live births) (Hennekam, 2006) or the more common syndrome Saethre-Chotzen/acrocephalosyndactyly type III (1:25,000-1:50,000 live births) (Rubinstein and Taybi, 1963; von Gernet et al., 1996). These two syndromes are difficult to distinguish because both are caused by reductions in either CBP or Twist function, and both are characterized by craniofacial and digit dysmorphologies. Importantly, the identification of the molecular underpinnings of these developmental abnormalities illustrates how the search for defects in specific developmental genes has become a vital and quickly evolving field in medical genetics (Harper, 2010).

#### The decapentaplegic/bone morphogenetic protein signaling pathway

The transforming growth factor β (TGF-β) superfamily comprises a large group of structurally related secreted signaling molecules that belong – based on similarities in sequence and function – to three subfamilies: the bone morphogenetic proteins (BMPs), the activin/inhibins, and the TGF-β proteins (Attisano and Wrana, 2002). TGF-β superfamily members signal through conserved transmembrane serine/threonine kinase receptor complexes, with signals transduced intracellularly via phosphorylation and activation of Smad transcription factors (Massague, 2012). TGF-β superfamily members play essential roles in embryonic patterning and tissue morphogenesis that are conserved among metazoans (Wu and Hill, 2009). As an example, bone morphogenetic protein 4 [BMP4; called Decapentaplegic (Dpp) in *Drosophila*] has numerous conserved roles during embryonic patterning and development: in the dorsoventral (DV) axis, and in the eye, heart and otic vesicle (Chen et al., 2004; Pujades et al., 2006; Slavotinek, 2011; Wall and Hogan, 1994). Given this conservation in function, it is not surprising that the phenotypic consequences of abnormal Dpp signaling in *Drosophila* bear similarities to human developmental disorders in which the orthologous BMP pathway is disrupted.

Flies provide an important experimental model in which to discern the mechanism and etiology of BMP4-associated human developmental disorders. These include **anophthalmia/microphthalmia**, microphthalmia syndromic 6 orofacial cleft 11, and **brachydactyly** type A2 (Bakrania et al., 2008; Lehmann et al., 2006; Suzuki et al., 2009). Eye, palate and digit defects, respectively, feature prominently in the clinical manifestation of these syndromes, and thus it is clear that BMP4 signaling deficiencies in humans are associated with an array of developmental defects identical to those already well-documented for Dpp in *Drosophila* (Simin et al., 1998; Spencer et al., 1982). Moreover, at the level of biological process, *Drosophila* Dpp signaling patterns the early embryo and **imaginal discs** (O'Connor et al., 2006), and regulates actin rearrangements that underlie the zippering of epithelial sheets during the essential embryonic process of dorsal closure in *Drosophila* (Glise and Noselli, 1997; Martin and Wood, 2002). Thus, as Twist transcriptional activity is required for proper mesoderm differentiation in both flies and humans, so also is Dpp/BMP signaling activity required for conserved developmental processes in flies and humans. Dpp/BMP conservation extends from the molecular level to that of biological process, demonstrating that mechanistic insights into developmental events made in flies can be extended to humans.

The identification of Dpp pathway antagonists in flies (Campbell and Tomlinson, 1999; Francois et al., 1994; Humphreys et al., 2013; Shimell et al., 1991) has revealed that increased levels of Dpp/BMP signaling also have lethal developmental consequences, contributing substantially to our understanding of the rare, but devastating, autosomal dominant ectopic bone formation disorder fibrodysplasia ossificans progressiva (FOP; 1:2,000,000 live births) (Pignolo et al., 2013). The most common mutation underlying this condition is R206H in the glycine-serine (GS) activation domain of the BMP type 1 receptor ACVR1 [called Saxophone (Sax) in *Drosophila*] (Shore et al., 2006). This missense mutation leads to constitutive ACVR1 activation and increased phosphorylation of downstream targets, including the transcription factor Smad1 (van Dinther et al., 2010). Discoveries made in *Drosophila* concerning the architecture of this pathway have provided a foundation for drug studies into kinase inhibitors as potential therapeutics for treating FOP (Kaplan et al., 2013, 1990; Le and Wharton, 2012; Twombly et al., 2009). Excessive TGFβ signaling also provides the foundation for our understanding of osteogenesis imperfecta, a heritable disease in which altered TGF-β signaling is thought to affect bone quantity and quality and thus result in bone fragility (Grafe et al., 2014).

#### The Hedgehog/Sonic hedgehog signaling pathway

Our understanding of the Hedgehog (Hh) signaling pathway [called Sonic hedgehog (SHH) in mammals], and how it contributes to congenital conditions, also has its foundations in *Drosophila* genetics. The Hh receptor, encoded by the gene *patched* (*ptc*; *PTCH1* in humans), was first identified in the Heidelberg screens for lethal patterning defects (Nusslein-Volhard and Wieschaus, 1980) and was subsequently cloned (Hooper and Scott, 1989; Nakano et al., 1989). Many other components of the Hh pathway were also identified in *Drosophila*, based on their similar loss-of-function embryonic phenotypes, well before their mouse orthologs were identified and cloned (Goodrich et al., 1996; Hahn et al., 1996). The observation that animals (both flies and mice) homozygous for loss-of-function Hh/SHH pathway mutations die in embryogenesis provides strong evidence that this signaling pathway fulfills conserved developmental roles. Decreased SHH signaling (either through haploinsufficiency for *SHH* or by increasing the repressive activity of PTCH1) has severe developmental consequences that mirror human holoprosencephaly (HPE), a common forebrain defect resulting from the failure of the cerebral hemispheres to separate. Few HPE fetuses survive to birth, but nonetheless the disorder is diagnosed in 1:20,000 live births (Edison and Muenke, 2003).

Although most HPE cases are considered sporadic, familial cases have also been described (Heussler et al., 2002). The most commonly mutated gene in both sporadic and familial forms of the disease is *SHH*, but mutation of other pathway components (for example, in the receptor *PTCH1*, and in a SHH target gene, the transcription factor *GLI2*) have also been causally linked to the disorder (Ming et al., 2002; Ming and Muenke, 2002; Roessler et al., 2003). The pathway has long been known to be essential for forebrain patterning (Hebert and Fishell, 2008). The lack of clear genotype-to-phenotype correlations associated with HPE (Traiffort et al., 2004) underscores our recognition that most genetic diseases, including HPE, are complex. This complexity is usually interpreted to mean that genes do not act in isolation, but rather in concert with their individual genetic backgrounds and/or environments. In cases like this, *in vivo* modifier screens and quantitative (high-throughput) functional genomic assays in cell culture are invaluable for a comprehensive understanding of pathways as well as for a fuller understanding of loci contributing to dysmorphic disease susceptibility in the long term (St Johnston, 2002). Indeed, both types of second-generation screens have yielded conserved modulators of Hh/Shh pathway activity, including the phosphoprotein phosphatase Microtubule star (Mts) and the cell-surface glypican Dally-like (Dlp) (Casso et al., 2008; Lum et al., 2003).

Interestingly, many genes associated with developmental defects are also linked to neoplasia. For example, mutations in SHH signaling pathway genes cause autosomal dominant basal cell nevus syndrome (BSNS) (Hahn et al., 1996; Johnson et al., 1996), a condition defined by a wide range of clinical manifestations, including the development of postnatal skin tumors in association with malformations of the ribs (duplicated, fused, splayed or misshapen) and skull (especially its enlargement) (Gorlin and Goltz, 1960). Such overgrowth phenotypes are now better understood in light of discoveries made in *Drosophila* on the role of Hh as a negative regulator of cell growth and proliferation (Ingham, 1998; Neumann, 2005).

#### The Notch signaling pathway

Several human congenital disorders are associated with mutation of the Notch pathway. John Dexter and Thomas Hunt Morgan described the first *Notch* alleles (in flies with notched wings) almost 100 years ago (Morgan, 1917). The Artavanis-Tsakonis and Young labs independently cloned and sequenced the *Drosophila* gene (Kidd et al., 1986; Wharton et al., 1985), paving the way for additional mechanistic studies in flies and worms. The *Notch* gene encodes a transmembrane receptor that is proteolytically cleaved upon ligand binding, with the cleaved intracellular domain entering the nucleus to regulate gene expression (Greenwald, 2012; Lieber et al., 1993; Struhl and Adachi, 1998; Struhl et al., 1993; Struhl and Greenwald, 2001). The conserved Notch pathway is one of the most widely used mechanisms of intercellular communication in all metazoan organisms, and a century of work deciphering the developmental roles of Notch signaling in *Drosophila* has provided the basis for more recent insights into the central role of the Notch pathway in human development (Yamamoto et al., 2014).

In humans, loss of function of the Notch2 receptor or of its ligand Jagged leads to Alagille syndrome, an autosomal dominant condition that is moderately prevalent, with an occurrence of 1 in 20,000 live births (Kamath et al., 2003). The syndrome is distinguished by bile duct paucity; in addition, abnormalities of the heart, eye and skeleton often occur in association with distinctive facial features (Kamath et al., 2003). Importantly, bile duct epithelial morphogenesis defects displayed by individuals with Alagille syndrome and *Notch^Δ/+^; Jagged^Δ/+^* double-heterozygous mice are reminiscent of the epithelial morphogenesis defects observed in Notch pathway *Drosophila* mutants (Hartenstein et al., 1992).

More generally, the Notch signaling pathway plays a conserved role in organ development in all metazoa – ranging from insect to nematode to echinoderm to human; effects of pathway mutation are pleiotropic and dependent on dose and context (Gridley, 2003). Additional congenital disorders associated with defects in Notch signaling include **spondylocostal dysostosis** (a skeletal disorder), **lateral meningocele syndrome** (LMNS; a disorder distinguished by craniofacial dimorphism), **CADASIL** (a vascular disorder) and **bicuspid aortic valve disease** (a malformation of the aorta) (Chapman et al., 2011; Garg et al., 2005; Gripp et al., 2015; Rutten et al., 2014). Hyperactivation of the pathway can also lead to developmental abnormalities, e.g. **infantile myofibromatosis-2** (IMF2; a disorder of mesenchymal proliferation) (Martignetti et al., 2013). The Notch pathway loss-of-function phenotypes that are shared between flies and humans, e.g. epithelial morphogenesis (described above) and embryonic neurogenesis (de la Pompa et al., 1997), highlight the conserved roles for Notch signaling in development and further emphasize the power of insect models for probing mechanisms of human development.

### Forward genetics – gleaning insights into tissue morphogenesis

In accordance with their developmental roles in *Drosophila*, mutations in several human genes cause predictable, analogous defects. For example, in both flies and humans, mutations in HOX genes and Hox family members alter spatial identities: mutations in the Hox family member *Pax6* [called *eyeless* (*ey*) in *Drosophila*] eliminates eyes; mutations in *SALL1* [which has two homologs in *Drosophila*, called *spalt major* (*salm*) and *spalt-related* (*salr*)] disrupt eye and auditory elements (respectively); and mutations in *Nkx2-5* [called *tinman* (*tin*) in *Drosophila*] lead to heart defects. In all cases, these genes encode transcription factors that are components of conserved **gene regulatory networks** (GRNs; genomic subsystems that coordinate inputs from transcriptional activators and repressors during differentiation and development). Importantly, GRNs are evolutionarily conserved in their transcriptional regulation of similar sets of effector genes. Thus, the organ and tissue systems that flies share with humans are not only functionally analogous but also constructed from similar building blocks. The depth of this homology validates the use of fly models to provide detailed portraits of human tissue and organ development.

Below we discuss three model *Drosophila* biological systems (eye, heart and lung) that illustrate how forward genetic methods have been useful not only for organizing human developmental disorders on the basis of signal transduction pathways, but also for validating models of development on the basis of conserved complex GRNs.

#### Eye development

Although long thought to exemplify convergent evolution, the *Drosophila* compound and mammalian camera eyes have actually diverged in evolution (Gehring, 2014). In both flies and mammals, the eye is the product of the Pax6 (Ey in the fly) master regulator, a **homeodomain transcription factor** conserved in evolution (Quiring et al., 1994). *ey* is both necessary and sufficient to specify eye development in flies, and the human homolog functions heterologously to direct the making of an eye in flies (Halder et al., 1995). Moreover, loss of *Pax6* or additional components of the eye GRN produces aniridia (iris hypoplasia) not only in flies and humans, but also in zebrafish, frogs, chicks and mice (Bhatia et al., 2013; Kaufman et al., 1995; Nakayama et al., 2015; Takamiya et al., 2015; Treisman, 1999). In line with this, Gehring and colleagues demonstrated that the transcription factors encoded by *Pax2* (called *D-Pax2* or *shaven* in *Drosophila*) and *Sox2* (called *SoxN* in *Drosophila*), along with the lens-specific DC5 enhancer (defined in chick), form a conserved regulatory circuit responsible for secretion of crystalline, an essential lens protein (Blanco et al., 2005). Thus, conserved downstream effectors of GRNs function in specialized cells of the eye, and the effects of master regulators are properly parsed. There is a wide-ranging literature focused on Pax6 function in eye development (Gehring, 2002; Gehring and Ikeo, 1999; and references therein), and it is clear that the *Drosophila* genetic system has provided a particularly informative model in which to study the development of visual systems in compound and camera eyes alike (Pennisi, 2002; Pichaud and Desplan, 2002).

The eye is one the best-studied tissues in *Drosophila*, with a wealth of knowledge coming from high-throughput studies of genes with loss-of-function phenotypes in the eye that are easily visualized using reflected light and/or scanning electron microscopy (Baker et al., 2014). Several standard forward genetic screens have been performed to identify genes required for eye development (e.g. Janody et al., 2004; Moberg et al., 2001; Tapon et al., 2001), whereas modifier screens (Box 2), dependent upon dose-sensitive perturbations of development, have been used in especially elegant ways to study the fundamentals of receptor tyrosine kinase and Ras signaling (e.g. Karim et al., 1996; Rogge et al., 1991; Simon et al., 1991).

#### Heart development

The heart, like the eye, is ancient in origin, with its development controlled by an evolutionarily conserved GRN. In *Drosophila*, the heart is known as the dorsal vessel and it functions as a linear peristaltic pump. Each of the core GRN elements required to enact the cardiac genetic program in humans is also expressed in the *Drosophila* heart. All core GRN elements are transcription factors: NKX2 (at least two in humans; Tinman in *Drosophila*); MEF2 and Hand (both known by the same name in *Drosophila*, with two and four homologs, respectively, in humans), GATA (three homologs in humans; Pannier in *Drosophila*); and Tbx (at least seven homologs in humans: Midline and H15 in *Drosophila*) (Azpiazu and Frasch, 1993; Bodmer, 1993; Han et al., 2006; Kolsch and Paululat, 2002; Miskolczi-McCallum et al., 2005; Reim et al., 2005; Sorrentino et al., 2005). *MEF2*, which is conserved from yeast to humans, encodes the most ancient myogenic transcription factor on record (Potthoff and Olson, 2007). It is expressed in the cardiac structures of flies and humans, as well as in all organisms lying between them in the evolutionary spectrum (Black and Olson, 1998).

In humans and flies, mutations in any component of the heart GRN lead to congenital heart disease, the most common birth defect in humans (Global Burden of Disease Study 2013 Collaborators, 2015). Notably, mutations of the human NK2 family member NKX2 homeobox 5 (*NKX2-5*) are associated with cardiac conduction abnormalities, as well as ventricular and atrial septal defects (Elliott et al., 2003); in the fly, *tinman* mutants lack the dorsal vessel. The mechanisms by which the loss of GRN transcription factors TBX5 and TBX1 can lead to inborn errors of development (Holt Oram syndrome and cardiac outflow tract abnormalities, respectively), has been particularly well studied in model systems, including *Drosophila* (e.g. Fink et al., 2009; Porsch et al., 1998; Schaub et al., 2015).

A lack of **genetic redundancy** in the fly has been particularly important for advancing our understanding of heart development because it allows phenotypes to be seen in single mutants that would not otherwise be detectable in higher eukaryotes, which have greater redundancy (Olson, 2006). Several moderate- to high-throughput tools have been developed that allow investigators to probe models of heart disease in the fly (Ugur et al., 2016). First, we are equipped to view the *Drosophila* larval and pupal beating hearts using a standard dissection microscope for analysis (Cooper et al., 2009; Wessells and Bodmer, 2004). Second, a more sensitive, but lower throughput, methodology to assess heart function in fixed samples is optical coherence tomography (OCT), a 3D subsurface imaging technique (Bradu et al., 2009). Finally, relying on genetic methods of analysis, we can employ heart-specific *GAL4* drivers (like *tinman:GAL4*) to express GFP in the hearts of mutants, and conventional **epifluorescence** (or confocal microscopy as a backup) for real-time observation of heart function (Lo and Frasch, 2001; Qian et al., 2008).

#### Lung development and branching morphogenesis

Insights into the genetic control of lung epithelial outgrowth (also known as branching morphogenesis) have their foundation in traditional loss-of-function studies of *Drosophila* (Baer et al., 2007; Chanut-Delalande et al., 2007; Ghabrial et al., 2011). The *Drosophila* tracheal system comprises a network of tubes that lead from openings on the surface of the animal and subdivide into smaller and smaller tubes that deliver oxygen to internal tissues (Behr, 2010). The primary branches of the tracheal system are set down during embryonic development, deploying genetic programs similar to those functioning in human lung development (Liu et al., 2003). The simple structure of the *Drosophila* respiratory system makes it particularly appealing as a prototypical model for studying branching morphogenesis. Respiratory development begins with the formation of small bud-like sacs, a process dependent on two genes [*trachealess* (*trh*) and *tango* (*tgo*)] that each encode a basic helix-loop-helix (bHLH) protein (for which vertebrate counterparts remain unidentified). The subsequent elongation (in both flies and humans) of these branches depends on the Sprouty and FGF proteins, with Sprouty negatively regulating FGF10 [called Branchless (Bnl) in *Drosophila*] (Hacohen et al., 1998; Warburton et al., 2001). *Drosophila bnl* mutants have airways that are wider and shorter than normal (Jarecki et al., 1999); in mammals, loss of the FGF10 receptor FGFr2b [Breathless (Btl) in *Drosophila*] is incompatible with viability, producing undifferentiated epithelial tubes (Gredler et al., 2015; Mailleux et al., 2002).

At the end of *Drosophila* embryonic development, specialized cells within the tracheal system, called terminal cells, undergo dramatic morphogenetic changes by extending numerous thinly branched cytoplasmic projections (Ghabrial et al., 2003). Terminal cell branching is exquisitely sensitive to oxygen physiology, both in target tissues and in the terminal cells themselves (Jarecki et al., 1999). In addition, terminal cell branching is readily quantifiable. Assessment of the effects of genetic mutations on terminal cell development has revealed terminal-cell-autonomous and non-autonomous requirements for oxygen (Ghabrial et al., 2011). *Drosophila* models have also been used to test for genes associated with congenital lung disease such as asthma (e.g. *Tl*; Roeder et al., 2012), and congenital lung defects such as airway remodeling (e.g. *rhomboid*; Affolter et al., 2003) and tubulogenesis (e.g. *unpaired*; Maruyama and Andrew, 2012).

### Reverse genetics – genotype-to-phenotype considerations

Developmental pathways are deeply conserved, indeed to the extent that they are considered universal (Halder et al., 1995); thus, our understanding of developmental processes in humans can be informed by an understanding of orthologous gene functions in model organisms. Recent improvements to and wide applicability of reverse genetic strategies to systematically target gene inactivation (Hardy et al., 2010) now makes it possible to expeditiously assess the roles of orthologs of human disease gene candidates in models systems such as the fly (see Fig. 1).

The Human Genome Project was a landmark endeavor, undertaken with a clear imperative to galvanize the field of medical genetics by supporting the diagnosis and management of hereditary disorders. With the sequence of the human genome now available, we must now consider how to link DNA sequences to the emergent properties of that genome. However, although genome annotation challenges have been embraced and automated, we have fallen behind in our ability to analyze at the functional level the tremendous amount of available genomic data. This is the genotype-to-phenotype bottleneck. Put another way, the speed of discovery of rare disease-causing genes has outpaced our ability to understand mechanistically how mutant alleles lead to clinical symptoms and disease. Addressing this challenge requires the development, characterization and sharing of new animal models of human disease.

**Fig. 2.**
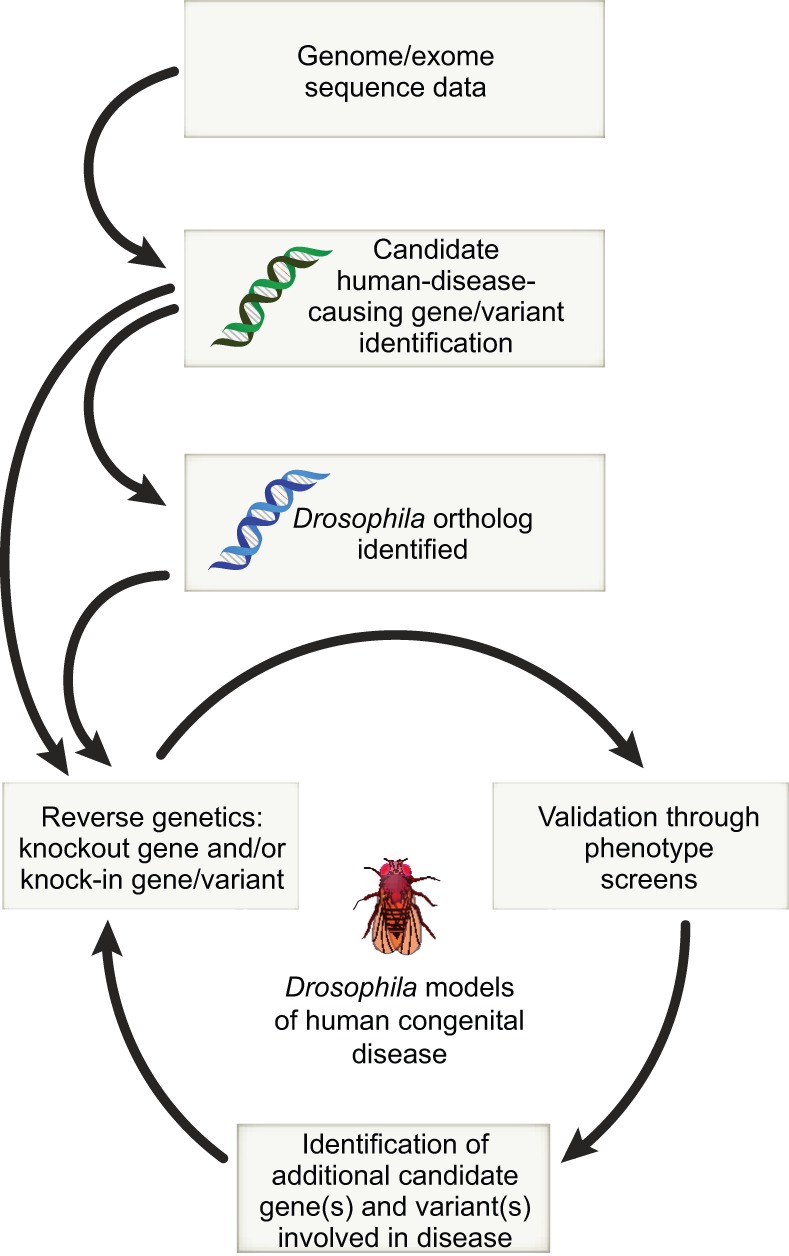
**The *Drosophila* pipeline for modeling human disease.** Candidate disease-causing mutations are identified using variant sequence data obtained from patient sources, including whole-genome and exome sequence datasets. When *Drosophila* orthologs of candidate disease-causing genes are identified, they can be targeted for disruption and/or a human gene variant can be introduced into the fly genome; phenotypic studies are used to assess validity of the model. Upon validation, fly models of human disease and development can be used as screening platforms for the identification of additional genes and variants involved in the conserved disease/development process, and for the identification of drugs and therapies.

The OMIM (Online Mendelian Inheritance in Man) database is a valuable resource that can point the translational scientist to rare congenital disease candidate genes that have likely orthologs in *Drosophila*, with the expectation that these orthologs can be interrogated in insect models, even without prior assignment to a biological pathway. As a starting point, Hu and colleagues (2011) used MeSH (Medical Subject Heading) terms to identify 2283 *Drosophila* genes that share at least one functional annotation with a human ortholog associated with a disease. Their analysis confirms our expectations that genes conserved functionally at the biochemical level are frequently also conserved at the biological level, and illustrates how the identification of orthologs can be an important first step to using a *Drosophila* model (or indeed any animal model) to study human congenital disease (Fig. 2). Conserved genotype-phenotype relationships in flies and humans are vital to the success of reverse genetic strategies, allowing us to make accurate predictions about loss-of-function phenotypes in *Drosophila* for orthologs of human disease candidate genes, the obligatory first step in human disease modeling. In line with this, FlyBase recently introduced Human Disease Model Reports, an integration of disease-related information from different databases (including OMIM). These reports provide a universal/less-specialized entry point for both *Drosophila* and non-*Drosophila* researchers interested in fly models of disease (Millburn et al., 2016).

Our ability to manipulate the fly genome has progressed in line with advances in discovering disease-causing mutations. These technological developments have allowed us to interrogate human disease candidate gene functions in *Drosophila* using reverse genetic approaches. One expedient way to do this is through the use of temporally and spatially controlled RNA interference (RNAi) using the UAS:GAL4 system (Box 2). This combinatorial approach makes it possible to disrupt gene activity at a level of resolution that was difficult to achieve when only classical genetic loss-of-function methods were available. Current state-of-the-art methodology exploits a set of double-stranded RNAs (dsRNAs) to achieve genome-wide RNAi knockdown. By fusing an inverted tandem repeat DNA sequence to the yeast-derived *UAS* promoter, dsRNA expression can be controlled in trans through the temporal- and/or tissue-specific expression of yeast *GAL4*. CRISPR/Cas9 genome-editing techniques (Box 2) offer unique opportunities to precisely recreate loss-of-function mutations *in situ* (Gratz et al., 2015a,b); however, there are no current reports of disease models that take advantage of this genome-editing technique in the fly.

The *Drosophila* RNAi Screening Center (DRSC) at Harvard University has undertaken an effort to generate and utilize RNAi constructs for various research applications. With the aim of understanding the function of genes suspected of causing **orphan human diseases**, the DRSC has generated more than 9000 UAS:RNAi transgenic fly lines (designated TRiP for Transgenic RNAi Project), 1575 of which target the *Drosophila* orthologs of human genes linked to disease (Hu et al., 2011). Notably, the TRiP RNA collection provides 85% coverage for 670 high-confidence disease-associated human genes with similarly high-confidence *Drosophila* orthologs (http://www.flyrnai.org/HuDis). TRiP lines are readily available (from DRSC and Bloomington Stock Center). The Vienna *Drosophila* RNAi Collection (VDRC) currently boasts a set of almost 32,000 *Drosophila* transgenic RNAi lines, corresponding to an estimated 90% of the entire fly genome (Dietzl et al., 2007). Although the VDRC collection is larger than the TRiP collection, fewer of the RNAi lines that it contains are the product of targeted integration, and evidence suggests that validated phenotypes are more readily obtained with the use of TRiP lines (Green et al., 2014). Taken together, though, these resources ensure a human congenital disease validation pipeline in *Drosophila* (with some examples briefly described here) that is less costly and less time consuming than reverse genetic validation strategies in vertebrate model systems (Bell et al., 2009; Giacomotto and Segalat, 2010). Although these and other genetic tools (Table 2) are unmatched in any other model system, invertebrates might not always provide exact models of human development and there are known human disease genes for which there is no fly ortholog (Chien et al., 2002; Reiter et al., 2001). In these cases, a vertebrate model system might be better suited for analysis.

**Table 2.**
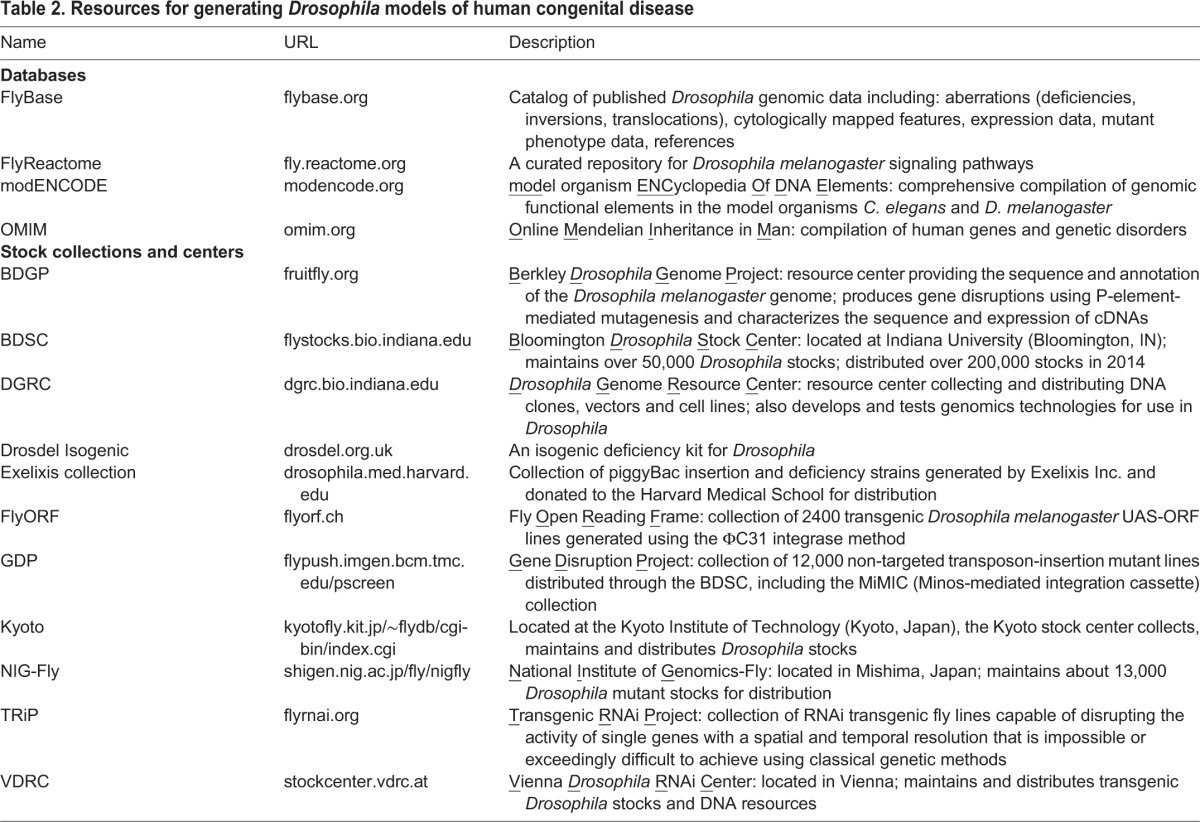
**Resources for generating *Drosophila* models of human congenital disease**

Human and *Drosophila* sequence databases, in combination with emerging compilations of phenotype annotations in both species, are the large 21st century datasets that serve as a starting point for reverse genetic strategies to generate *Drosophila* models of human congenital disorders, some of which are described below.

#### Hypoparathyroidism-retardation-dysmorphism syndrome

Hypoparathyroidism-retardation-dysmorphism (HRD) syndrome, which is also diagnosed as Sanjad-Sakati or Richardson-Kirk syndrome, is a rare, autosomal recessive inherited condition characterized by congenital hypothyroidism, mental retardation, and growth failure associated with facial dysmorphia (Abdel-Al et al., 1989; Richardson and Kirk, 1990; Sanjad et al., 1991). HRD results from mutations in the *TBCE* (tubulin-specific chaperone E) gene, which encodes a protein that is required for the proper folding of alpha-tubulin subunits and thus for the formation of alpha-beta-tubulin heterodimers (Parvari et al., 2002). The mechanism by which mutated *TBCE* causes HRD is not well understood. *Drosophila* geneticists seeking to generate a fly model of HRD identified by bioinformatics analysis one high-scoring *Drosophila TBCE* ortholog, *tbce*, for which they generated RNAi targeting constructs, as well as classic **amorphic alleles** (Jin et al., 2009). *Drosophila tbce* mutants exhibit a range of phenotypes, including abnormalities in microtubule distribution that are reminiscent of human HRD phenotypes and which are shared by individuals with related conditions, including fragile X syndrome (FXS) and hereditary spastic paraplegia (Sherwood et al., 2004; Trotta et al., 2004; Zhang and Broadie, 2005). The *Drosophila* model has proven especially useful for studying the molecular pathogenesis of HRD: genetic tests of **epistasis** have led to the identification of spastin (itself linked to hereditary spastic paraplegia) as a TBCE partner in microtubule regulation (Jin et al., 2009), providing translational scientists with new insights into TBCE's mechanism of action.

#### CHARGE syndrome

RNAi silencing and targeted gene-disruption approaches in *Drosophila* are also being used to model CHARGE syndrome, a common autosomal dominant disorder (1:10,000 live births) associated with wide-ranging congenital dysmorphologies, including malformations of the nasal cavity, heart, inner ear and retina (Blake et al., 1998). Two thirds of CHARGE syndrome cases are caused by mutations in the chromatin-organizing protein chromodomain helicase DNA-binding gene 7 (*CHD7*; called *Kismet* in *Drosophila*) (Sanlaville and Verloes, 2007). However, the role of CHD7 in generating the array of congenital anomalies seen in individuals with CHARGE syndrome remains unclear. The *Drosophila* model recapitulates several important aspects of the human disease (Ghosh et al., 2014; Melicharek et al., 2010), but a greater understanding of how the animal model might be best exploited to understand CHARGE syndrome perhaps comes from studies of loss-of-function mutants in *Drosophila* chromatin-organizing proteins belonging to the Polycomb group (Duncan and Lewis, 1982). In these mutants, loss of chromatin organization leads to the dysregulation of homeotic gene targets and results, not surprisingly, in wide-ranging developmental deficiencies.

#### Treacher Collins syndrome

Treacher Collins syndrome (1/50,000 live births) is an autosomal dominant craniofacial dysmorphology disorder caused by mutations affecting the protein TCOF1 (Treacher Collins-Franceschetti syndrome 1; Nopp140 in *Drosophila*). 60% of cases occur in infants with no previous family history of the disease, and are thus thought to arise *de novo*. Treacher Collins syndrome has been successfully modeled in flies through the disruption of *Nopp140*, which encodes a 140-kDa nucleolar and Cajal body phosphoprotein that is thought to be a ribosome assembly factor, although its specific function remains unknown (Waggener and DiMario, 2002). Whereas complete loss of *Nopp140* function is incompatible with viability, a 30% gene disruption produces dysmorphologies in the wing, leg and tergite (Cui and DiMario, 2007). In addition, the *Nopp140^RNAi^* fly model has revealed how incomplete disruptions of Nopp140/TCOF1-dependent processes of nucleolar stress and cell death can lead to developmental dysmorphologies (He et al., 2015; James et al., 2013).

#### Congenital disorder of glycosylation, type IIc

Another example of the power of RNAi for generating *Drosophila* models of human congenital disease comes from studies of *Drosophila* Gfr (GDP-fucose transporter 1). In humans, mutations in *SLC35c1*, the human *Gfr* ortholog, cause the rare autosomal recessive congenital disorder of glycosylation, type IIc (CDG). Affected individuals exhibit severe mental retardation, short stature and characteristic facial dysmorphia, in addition to immune dysfunction (Frydman et al., 1992); oral administration of fucose alleviates postnatal immune deficiencies (Luhn et al., 2001). *Drosophila* geneticists, using RNAi-based knockdown strategies, discovered that flies exhibit Notch-like phenotypes when they lack Gfr and that Gfr is responsible for Notch *O*-fucosylation (Ishikawa et al., 2005). Given the previous association of the Notch pathway with Alagille syndrome, another congenital disorder associated with mental retardation, slow growth and facial dysmorphism (see earlier discussion of the Notch pathway), Ishikawa and colleagues interpreted their findings to mean that defective Notch signaling is responsible for the developmental defects associated with both CDG and Alagille syndrome. This study highlights how shared loss-of-function phenotypes generated by reverse genetic strategies can identify functional links between proteins, thereby advancing our understanding of human disease etiology and pointing us to improved diagnostic methods.

#### Townes-Brocks’ syndrome

Townes-Brocks' syndrome (TBS) is a rare autosomal dominant inherited malformation syndrome that is characterized by anal, renal, limb and ear abnormalities, and is uniquely associated with mutations in the *SALL1* gene, which encodes a transcription factor called Spalt-like 1 [Spalt major (Salm) in flies]. Flies null for *salm*, a target of the Dpp and Hh signaling pathways, suffer embryonic lethality (Jurgens, 1988). However, an analysis of the tissue-specific functions of *salm* and *spalt-related* (*salr*) in mosaic flies that carry both wild-type and mutant cells revealed that these flies manifest antennae and genitalia defects. In addition, electrophysiological assays confirm that these flies are also deaf (Dong et al., 2003). Thus, auditory and genital abnormalities in mutant flies are reminiscent of those seen in individuals with TBS, and our comprehensive genetic and molecular understanding of Sal regulatory circuits in flies can inform our understanding of the biological abnormalities associated with TBS in humans. In this regard, most disease-causing TBS alleles produce a truncated protein that, although able to correctly interact with other Spalt proteins (there are four in humans), is unable to function properly (de Celis and Barrio, 2009).

### Reverse genetics – humanized models

In addition to loss-of-function experiments dependent on forward and reverse genetic strategies, the versatile *Drosophila* experimental system also allows researchers to ‘knock-in’ genes of interest (usually gain-of-function alleles) using traditional transgenesis protocols. Most examples of the technique's utility for disease modeling in the fly comes from the analyses of neurodegenerative conditions, perhaps because these disorders share a common pathological denominator, protein misfolding. The subsequent formation of aberrant protein aggregates with toxic conformers selectively damage neuronal populations. In the case of Alexander disease, the autosomal dominantly inherited **leukodystrophy** is caused by mutations of *GFAP* (glial fibrillary acidic protein) for which there is no ortholog in flies. Nonetheless, glial expression of human mutant *GFAP* in transgenic flies induces the formation of Rosenthal fibers (inclusions that serve as markers of the human condition) and promotes glial-mediated neurodegeneration (Wang et al., 2011). Humanized *Drosophila* strains are used most widely to model neurological disorders (Bonini and Fortini, 2003; Jaiswal et al., 2012; Muqit and Feany, 2002), but also to study inborn errors of development, as we discuss below.

#### Noonan and LEOPARD syndromes

Mutation of *PTPN11*, which codes for the protein tyrosine phosphatase SHP2, is associated with two clinically related pleomorphic **RASopathies** (Noonan syndrome and LEOPARD syndrome), both of which are characterized by cardiovascular, craniofacial and skeletal malformations (Aoki et al., 2016). In the case of Noonan syndrome, gain-of-function missense mutations in *PTPN11* account for 50% of all cases, whereas mutations in other components of the **Ras/MAPK pathway** (*KRAS*, *SOS1* and *RAF1*) cause the remainder (Tidyman and Rauen, 2009). In all cases, gain-of-function missense mutations are thought to increase signaling through the Ras/MAPK pathway (Niihori et al., 2005). Noonan syndrome is inherited as an autosomal dominant disorder, but, for many affected individuals, there is no family history and cases are thought to result from *de novo* mutation. LEOPARD syndrome, which is also inherited in an autosomal dominant fashion and is distinguished from Noonan syndrome by the presence of multiple lentigines (café-au-lait spots), results only from a small set of *PTPN11* missense mutations, which are believed to be associated with the loss, rather than with the gain, of SHP2 function (Digilio et al., 2002).

In order to investigate how loss- and gain-of-function alleles of the same locus might lead to analogous phenotypes, *Drosophila* geneticists created transgenic flies that harbor the mutations found in the majority of individuals with LEOPARD syndrome {Y279C and T468M of the *PTPN11* gene [*corkscrew* (*csw*) in *Drosophila*]} to create humanized models of LEOPARD syndrome. Ubiquitous expression of either allele leads to ectopic wing venation and, in the case of Y279C, to rough eyes and increased numbers of the R7 photoreceptor – all readouts of increased RAS/MAPK signaling (Oishi et al., 2009). Recognition that LEOPARD syndrome mutations, despite their reduced src homology 2 (SH2) phosphatase activity, have gain-of-function developmental defects provided the first satisfying rationale for how *PTPN11* mutations with opposite effects on phosphatase activity might produce analogous phenotypes.

*Drosophila* transgenic models that harbor the gain-of-function *PTPN11/csw* mutations associated with either Noonan syndrome 1 (A72S and N308D) or juvenile myelomonocytic leukemia (E76K) (Oishi et al., 2006) have also been created; each mutation increases RAS/MAPK signaling, with A72S and E76K being the most active. Whereas ubiquitous expression of the two strongest alleles leads to embryonic lethality, expression of the Noonan-associated mutation N308D causes the formation of ectopic veins similar to those seen in the LEOPARD model.

The value of humanized allele models such as these should not be underestimated. They can be used to generate hypotheses that can then be tested in mammalian models, and provide a foundation for sensitized screens, which probe for mechanism through the identification of previously unknown interacting genes and/or therapeutic compounds. In recent years, *Drosophila* has gained traction as a repurposed tool to investigate congenital disorders of metabolism, such as diabetes (Jaiswal et al., 2012; Padmanabha and Baker, 2014), as well as syndromes caused by dominant mutations, such as the disorder epidermolysis bullosa simplex, a blistering skin disorder caused by dominant mutations in the keratin proteins keratin 5 or keratin 14 (Bohnekamp et al., 2015).

## Conclusions

The *Drosophila* embryo has been mined extensively, through classic genetic loss-of-function approaches, to advance our understanding of the fundamentals of development, including pattern formation, cell fate determination, morphogenesis and organogenesis. Indeed, as discussed in this Review, elegant combinations of genetics, molecular biology and biochemistry in the *Drosophila* embryo have been used to identify and characterize virtually every important signal transduction pathway in eukaryotes, from flies to humans. Now, when we identify *Drosophila* genes that have human orthologs suspected of having developmental roles, their specific functions can be assessed in high-throughput, embryonic-lethal-stage studies in *Drosophila*.

Some consider *Drosophila* to be multiple models rolled into one, with each of its life stages (embryo, larva, pupa and adult) offering unique opportunities to model human disease and development: the embryo is useful for the study of development; *Drosophila* larvae are useful for studying physiological processes and some simple behaviors (e.g. foraging); studies in pupae have been instrumental in investigating hormonal processes (e.g. Durisko et al., 2014; Huang et al., 2014; Nassel et al., 2013; Sokolowski, 2003; Weitkunat and Schnorrer, 2014) and the adult stage of the *Drosophila* life cycle can provide us with insights into neurodegenerative disease (Alzheimer's, Parkinson's, Huntington's, FXS), and sleep and seizure disorders, as well as into cognitive/psychosis and affective disorders, cancer, cardiovascular disease, inflammation and infectious disease, and metabolic diseases, including diabetes (for review see Pandey and Nichols, 2011; Alfa and Kim, 2016). Overall, the fly offers substantial opportunities for modeling human disease well beyond the congenital disorders we discuss here.

Of note too is our recognition that the fly response to drugs is oftentimes similar to that in mammals (Andretic et al., 2008; Satta et al., 2003; Wolf and Heberlein, 2003). One of the most important advances in model-systems drug discovery was centered on an analysis of small-molecule rescue of the fragile X phenotype in the *Drosophila* model of FXS (Chang et al., 2008). FXS, an X-linked dominant neurodevelopmental syndrome characterized by moderate to severe mental retardation, macroorchidism and distinctive facial anomalies, is caused by loss of the protein-synthesis inhibitor *FMR1* (fragile X mental retardation). *FMR1* mutation results from expansion of its CGG triplet, of which there are five to 40 repeats in wild-type alleles and 55 to 200 repeats in mutant alleles, and consequent silencing of the *FMR1* gene (Santoro et al., 2012). Both the neuronal and behavioral aspects of human FXS are recapitulated in flies, either through the targeted inactivation of the *Drosophila Fmr* gene or by overexpression of mutant alleles with various repeat lengths (Wan et al., 2000). Importantly, this fly model has been used successfully for drug discovery, with mGluR (a presumed FMR1 target) antagonists rescuing behavioral phenotypes in compound screens (Chang et al., 2008). mGluR studies have been extended successfully to mouse models of FXS (Dolen et al., 2010, 2007), although so far two different mGlu5 inhibitors have failed to benefit FXS patients in clinical trials (Scharf et al., 2015).

The fruit fly, with its genetic tractability and conserved genome, offers attractive and proven opportunities for gene validation and modeling of human developmental abnormalities, leading in the long term to 21st century precision medicine encompassing diagnostics and therapies. The many success stories highlighted in this Review provide compelling justification for expansion of methodologies in flies (as well as extension whenever possible to other models, including zebrafish and mice) to assess function of the candidate disease genes that are frequently identified in neonate whole-genome sequencing studies (Petrikin et al., 2015). The models that we discuss also highlight deep conservation in flies and humans that extends from genome sequence to biological process, providing a compelling argument for more frequent use of fly models in the drug discovery process. Although there are clear indications of success based on mechanistic insight for FOP (Kaplan et al., 2013; Le and Wharton, 2012) as well as compound screening for FXS (Chang et al., 2008), it is also clear that the fly represents an underutilized model in the drug discovery process.
